# Heparin and heparan sulfate proteoglycans promote HIV-1 p17 matrix protein oligomerization: computational, biochemical and biological implications

**DOI:** 10.1038/s41598-019-52201-w

**Published:** 2019-10-31

**Authors:** Antonella Bugatti, Giulia Paiardi, Chiara Urbinati, Paola Chiodelli, Alessandro Orro, Matteo Uggeri, Luciano Milanesi, Arnaldo Caruso, Francesca Caccuri, Pasqualina D’Ursi, Marco Rusnati

**Affiliations:** 1 Section of Experimental Oncology and Immunology, Department of Molecular and Translational Medicine, School of Medicine, University of Brescia, Brescia, Italy; 2 Section of Microbiology, Department of Molecular and Translational Medicine, School of Medicine, University of Brescia, Brescia, Italy; 3 Institute for Biomedical Technologies-National Research Council (ITB-CNR), Segrate, Milan Italy

**Keywords:** Polysaccharides, Infectious diseases

## Abstract

p17 matrix protein released by HIV+ cells interacts with leukocytes heparan sulfate proteoglycans (HSPGs), CXCR1 and CXCR2 exerting different cytokine-like activities that contribute to AIDS pathogenesis. Since the bioactive form of several cytokines is represented by dimers/oligomers and oligomerization is promoted by binding to heparin or HSPGs, here we evaluated if heparin/HSPGs also promote p17 oligomerization. Heparin favours p17 dimer, trimer and tetramer assembly, in a time- and biphasic dose-dependent way. Heparin-induced p17 oligomerization is of electrostatic nature, being it prevented by NaCl, by removing negative sulfated groups of heparin and by neutralizing positive lysine residues in the p17 N-terminus. A new computational protocol has been implemented to study heparin chains up to 24-mer accommodating a p17 dimer. Molecular dynamics show that, in the presence of heparin, two p17 molecules undergo conformational modifications creating a continuous “electropositive channel” in which heparin sulfated groups interact with p17 basic amino acids, promoting its dimerization. At the cell surface, HSPGs induce p17 oligomerization, as demonstrated by using B-lymphoblastoid Namalwa cells overexpressing the HSPG Syndecan-1. Also, HSPGs on the surface of BJAB and Raji human B-lymphoblastoid cells are required to p17 to induce ERK_1/2_ activation, suggesting that HS-induced oligomerization plays a role in p17-induced lymphoid dysregulation during AIDS.

## Introduction

Matrix protein p17 contributes to structural integrity of HIV virions and regulates viral replication^1,2^. Also, p17 is released by HIV-infected cells, being detectable in the nanomolar range in plasma, brain and lymph nodes of patients treated with HAART^3,4^. In its extracellular form, p17 deregulates the functions of B-lymphocytes^5,6^, contributing to AIDS progression and to the pathogenesis of AIDS-associated diseases^7,8^.

Heparan sulfate proteoglycans (HSPGs) consist of a core protein and of heparin-like glycosaminoglycan (GAG) composed of repeated disaccharides units of 2-O-sulfated L-Iduronic acid (IdoA) and N, 6-O-disulfated D-glucosamine (Glc) occasionally interrupted by non-sulfated uronic acids and under-sulfated hexosamines^9^. At the surface of leukocytes, they act as receptors for extracellular p17^10^. In the extracellular environment, free heparin released by mast cells sequesters p17, modulating its bioavailability^10^. Beside p17, HSPGs act as receptors also for HIV-1 gp120^11^ and Tat^12^ while free heparin promotes Tat oligomerization and biological activity^13^. HSPGs act as co-receptor also for many other viral proteins and cytokines, promoting their oligomerization required for receptors clustering and activation^14^.

p17 spontaneously oligomerizes forming trimers^15–17^ and even hexamers^18^. Two different domains have been identified in p17 that specifically mediate its self-assembling: the C-terminal region that self-interacts with the same region of other p17 molecules^19^, and amino acids E_42_-N_47_, Q_59_, Q_63_, that interact with the Q_69_-E_74_ region of another p17 molecule^20^. On the other hand, due to the presence of “coiled coil” sequences, p17 tends to misfold^21^, behaving as an “amyloidogenic” protein that forms toxic assemblies in the brain that have been demonstrated to contribute to AIDS-associated neurodegeneration^4^.

Taken together, the capacity of p17 to oligomerize and to bind to heparin/HSPGs, along with the involvement of the GAG in the process of oligomerization of many cytokines prompted us to study the effect of heparin/HSPGs on p17 oligomerization and biological consequences by adopting a multidisciplinary approach including bioinformatics, biochemical and cell-based models.

For what concerns bioinformatics, the docking of protein to heparin (used as a structural analog of HSPGs) and molecular dynamics (MDs) have been limited only to short (di-, tetra- or hexa-) oligosaccharides, mainly due to heparin conformational flexibility and high charge density, the weak surface complementarity of heparin/protein interactions, the absence of well-defined binding pockets, the difficulty to define the impact of solvation/desolvation, the large electrostatic interactions involved and the large number of torsional angles between glycosidic bonds^22,23^. Only in selected cases, 14-mer heparin oligosaccharides were used with molecular docking to study FGF^24^, VEGF^25^ and CXCL8^26^ dimerization. Relevant to this point, longer heparin or heparan sulfate (HS) chains are found in nature that are responsible for cytokine oligomerization. With these premises, to corroborate our experimental data, we have here modeled heparin chains up to 24-mer and performed docking and MDs with p17 dimer.

## Results

### Characterization of the spontaneous p17 oligomerization

**Figure 1:**
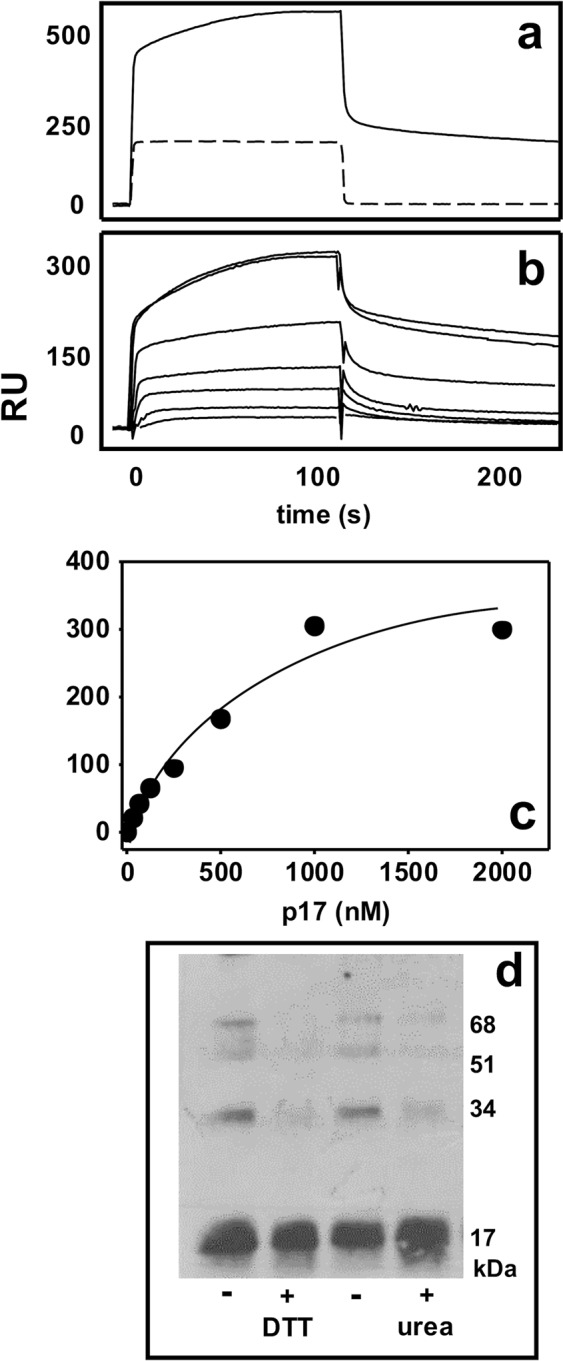
Spontaneous p17 oligomerization. (**a**) Sensorgrams showing the binding of free p17 to p17 immobilized to a sensorchip (straight line) or to a void sensorchip (dashed line). (**b**) Blank-subtracted sensorgrams overlay showing the binding of increasing concentrations of free p17 (from top to bottom: 2000, 1000, 500, 250, 125, 62.5, 31.2, 15,7 nM) to sensorchip-immobilized p17. (**c**) Saturation curve obtained by using the values of RU bound at equilibrium from injection of increasing concentrations of free p17 onto sensorchip-immobilized p17. (**d**) Representative WB analysis of cross-linked p17 oligomers after incubation in the absence or in the presence of DTT (1 mM) and UREA (8 M). In all the panels, the results shown are representative of other three-five that gave similar results.

p17 has been demonstrated to spontaneously self-assemble, forming trimers in solution^17^. We thus exploited SPR to calculate the kinetic parameters of spontaneous p17/p17 interaction. As shown in Fig. 1a, free p17 interacts with a p17-containing sensorchip but not with a void sensorchip (negative control). When increasing concentrations of free p17 are injected onto surface-immobilized p17, a sensorgrams overlay is obtained (Fig. 1b). Equilibrium binding data were then used to generate a saturation curve and Scatchard plot regression that allow the determination of a *Kd* value equal to 5.29 ± 1.5 × 10^−7^ M and 6.65 ± 1.8 × 10^−7^ M when using the softwares Prism GraphPad (Fig. 1c) and Origin Microcal (data not shown), respectively.

SPR analysis does not allow to discriminate among dimer, trimer or higher orders p17 oligomers. We thus used WB analysis of p17 after chemical cross-link, an approach that stabilizes and makes evident the different p17 complexes: p17 self-assembly gives origin to three order complexes: a dimer (the most abundant, corresponding to 29% ± 8,2 of total protein in the sample), a trimer (12% ± 3,0) and a tetramer (that corresponds only to 3% ± 0.03), in respect to a 59% ± 7.4 of the protein that remains in its monomeric form. Urea and dithiothreitol (DTT) prevent p17 oligomerization, indicating that a proper tridimensional conformation of the protein is required for self-assembly (Fig. 1d).

### Effect of heparin on p17 oligomerization

**Figure 2:**
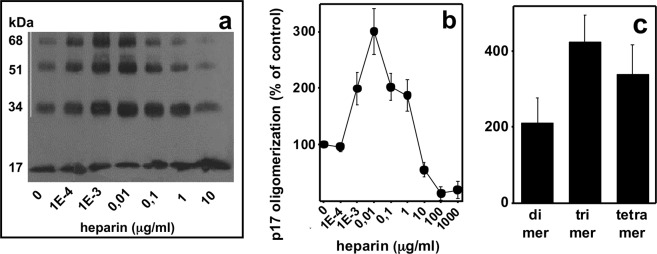
Effect of heparin on p17 oligomerization. (**a)** WB analysis of p17 cross-linked in the presence of increasing concentrations of heparin. The result shown is representative of four others that gave similar results. (**b**) Quantification of the cumulative intensity of the bands corresponding to p17 oligomers in the presence of increasing concentrations of heparin. (**c**) Quantification of the intensity of the bands corresponding to p17 dimer, trimer and tetramer in the presence of heparin (0,01 μg/ml). In panel b and c, data are expressed as % in respect to p17 oligomerization in the absence of heparin. The results shown are the mean ± S.E.M. of four independent experiments.

As already mentioned, by binding to proteins, heparin/HSPGs favour their oligomerization. Since heparin binds p17, we evaluated if its affects p17 oligomerization. Heparin modulates p17 oligomerization in a dose-dependent, biphasic way: at concentrations between 0.0001 and 0.001 μg/ml, it increases p17 oligomerization while, at higher concentrations (0.01–1,000 μg/ml) it causes an inhibitory effect (Fig. 2). When the formation of specific oligomers was considered, the stronger promoting effect is exerted by heparin on trimer and tetramer formation (4.2 and 3.4 fold increase), while dimer formation is increased only 2 times (Fig. 2c).

**Figure 3:**
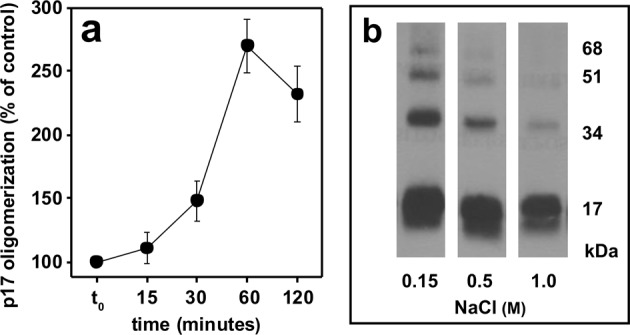
Characterization of heparin-induced p17 oligomerization. p17 was incubated with heparin (0,01 μg/ml) for the indicated period of time **(a)** or for 2 h in the presence of the indicated concentrations of NaCl **(b)**, cross-linked and analyzed in WB. In panel a, the cumulative intensity of the bands corresponding to p17 oligomers was quantified and expressed as % in respect to oligomerization in the absence of heparin (mean ± S.E.M. of three independent experiments). The result shown in panel b is representative of other two that gave similar results.

Heparin-dependent p17 oligomerization is time-dependent and relatively slow: to exert its full effect, heparin must be incubated with p17 for at least 60 min. (Fig. 3a). Also, heparin-dependent p17 oligomerization is ionic strength-dependent, being inhibited by NaCl (Fig. 3b).

**Figure 4:**
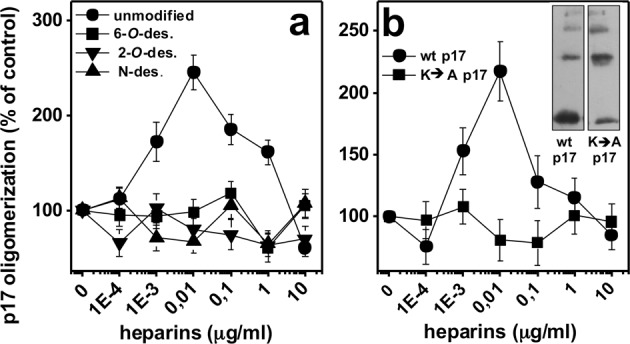
(**a**) Effect of selectively desulfated heparins on p17 oligomerization. p17 was incubated in the presence of increasing concentrations of unmodified heparin or of the indicated desulfated (des.) heparins, cross-linked and analyzed by WB. (**b**) Effect of heparin on the oligomerization of N-ter K → A p17 mutant. Wt or N-ter K → A p17 were incubated with the indicated concentrations of heparin, cross-linked and analyzed in WB. The cumulative intensity of the bands corresponding to p17 oligomers were then quantified and expressed as % in respect to oligomerization in the absence of heparin. The results shown are the mean ± S.E.M. of three independent experiments. Inset of panel b: representative WB analysis showing the spontaneous oligomerization of N-ter K → A p17 in the absence of heparin. A representative blot from a p17 oligomerization experiment performed with the wt protein is reported for comparison.

Heparin binds to p17 *via* its SO_3_^−^, which interact with the positive lysine residues of the N-heparin-binding domain (HBD) of the protein^10^. To evaluate if a direct p17/heparin interaction is required to p17 oligomerization, we exploited selectively 6-*O-*, 2-*O-* and *N*-desulfated heparins, already demonstrated to be unable to bind p17^10^: at variance with unmodified heparin, the three desulfated heparins do not promote p17 oligomerization, indicating that all the SO_3_^−^ of heparin are required to induce p17 oligomerization (Fig. 4a).

To characterize the features of p17 necessary for its heparin-dependent oligomerization, we used N-ter K → A p17, a mutant obtained by the substitution of lysine of the N-HBD with alanine (Fig. S1). N-ter K → A p17 shows a conserved capacity to undergo spontaneous oligomerization, indicating that the N-HBD is not involved in this process. Actually, for N-ter K → A p17 the spontaneous, basal oligomerization in the absence of heparin is even increased, possibly due to the neutralization of positive charges that, in wt p17, can limit self-interaction by electrostatic repulsion. However, heparin fails to promote N-ter K → A p17 oligomerization (Fig. 4b), in agreement with its incapacity to bind the protein^10^.

In conclusion, a direct interaction between the SO_3_^−^ of heparin and the positive lysine residues of p17 are required to induce protein oligomerization.

### Computational studies of heparin-induced p17 oligomerization

As already mentioned, computational docking of heparin (mostly used as a structural analogue of HSGPs) to its binding-protein still represents a challenge. Accordingly, the p17/heparin complex has been resolved only with a protein monomer and a 6-mer heparin^10^, prompting us to conceive a new computational approach to study the interaction of longer heparin chains with p17 dimers (Fig. S2).

Blind docking simulations with standard 4-mer heparin probes and wt p17 dimer show that the tetrasaccharides always take position in the N-HBDs of the two monomers. On the other hand, using a N-ter K → A p17 dimer, the 4-mer heparin probes dock mainly in the C-ter basic domain. We thus used a NC-ter K → A p17 dimer to predict alternative heparin-binding pose, allowing the definition of two alternative shorter and longer heparin paths.

**Figure 5:**
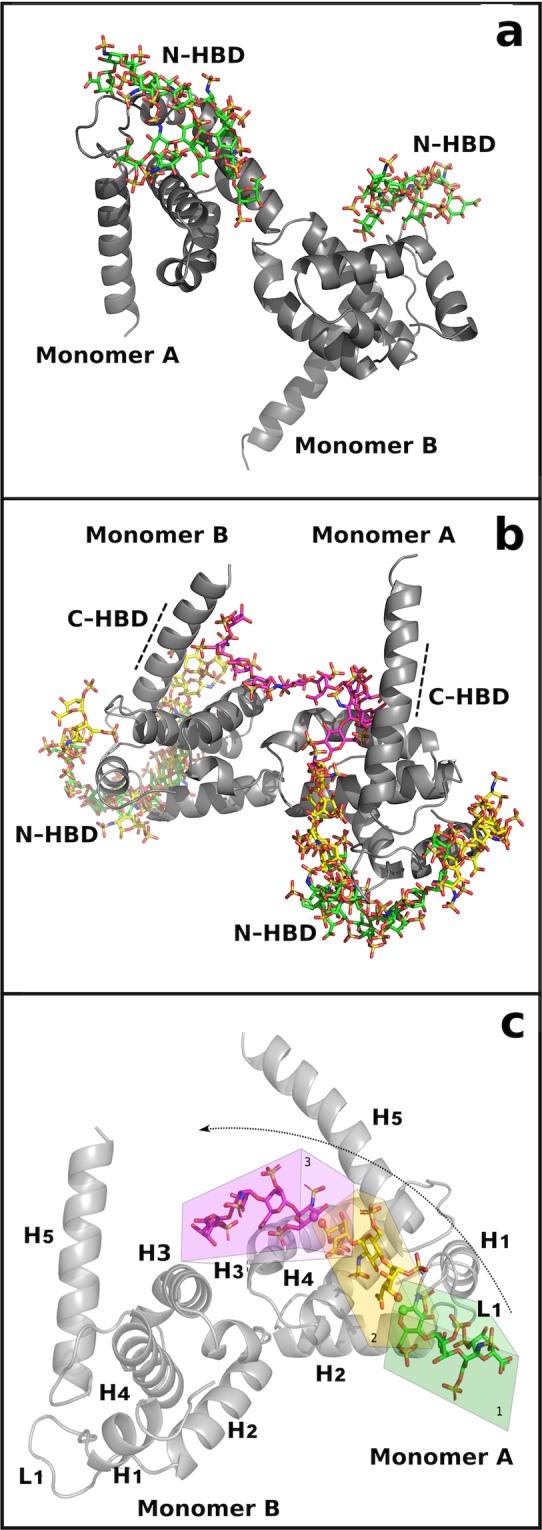
Shorter (**a**) and longer (**b**) heparin path identification by ClusPro web server. Position of the 4-mer heparin probes (in sticks) obtained by blind docking on the wt p17 dimer are in green, those obtained on N-ter K → A or NC-ter K → A p17 dimers are in magenta and yellow. p17 monomers are represented in grey cartoon. (**c**) Schematic representation of the sliding window method. Different regions composing the p17 protein are shown (H, α-helices; L, loops). The grid box of the first docked probe is in green, the second and third boxes, covering a whole 4-mer heparin and the last monomer of the precedent probe are in yellow and magenta, according to the heparin probes color. p17 monomers are represented as cartoon. For each heparin probe, in sticks, the atoms involved in 1 → 4 glycosidic linkages are represented as sphere.

In the shorter path, the succession of 4-mer heparin probes starts from the N-HBD of p17 monomer A and goes directly to the N-HBD of p17 monomer B (Fig. 5a). Also in the longer path the succession of 4-mer heparin probes starts from the N-HBD of monomer A but it makes then contact with H4, C-ter basic domain and H3 of monomer A and with H3, C-ter basic domain, H4 and finally N-HBD of monomer B (Fig. 5b).

**Figure 6:**
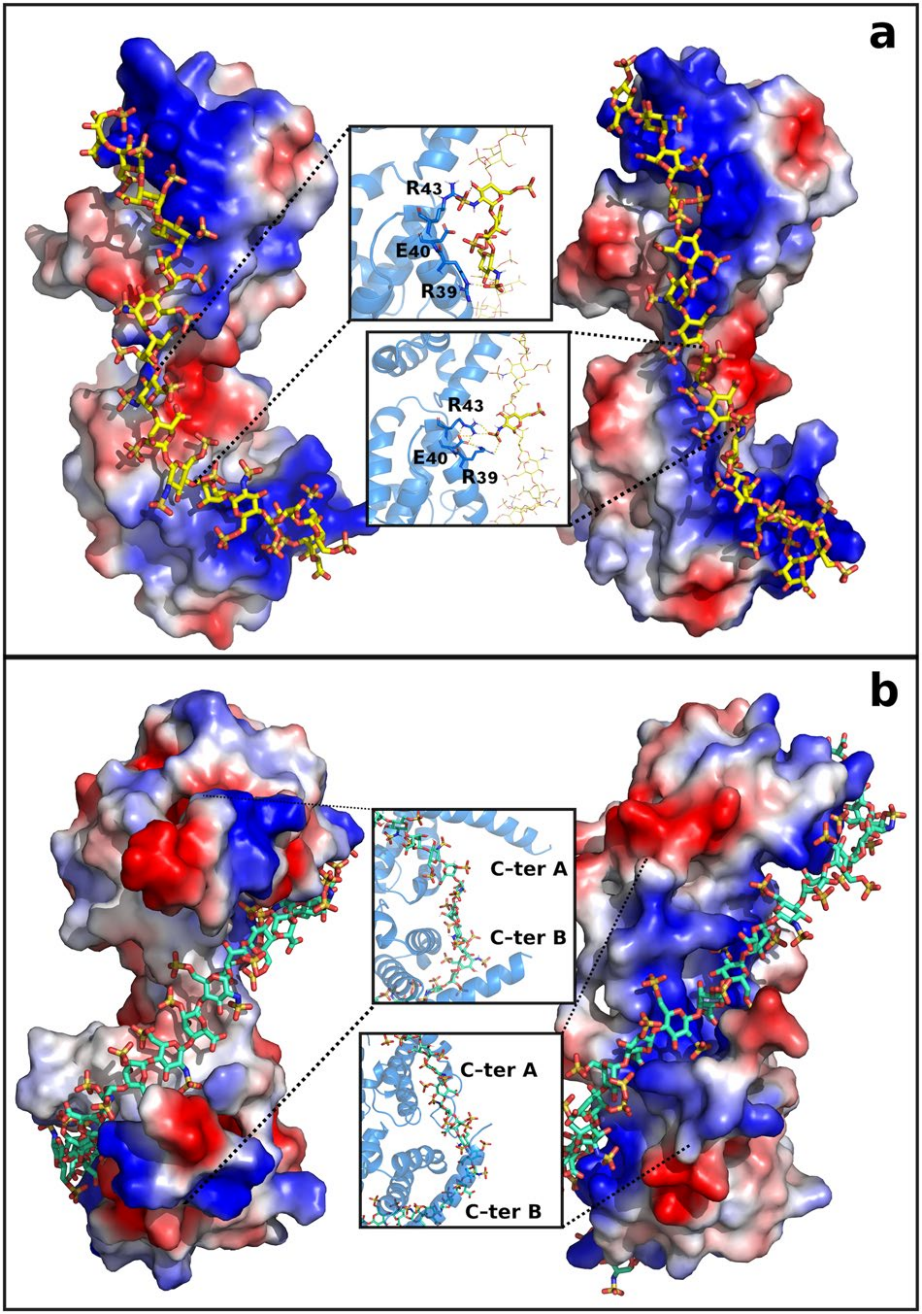
Electrostatic interactions. Docking prediction (left) and complex stability after MDs (right) of 15-mer **(a)** or 24-mer **(b)** heparin (yellow and green sticks for 15- and 24-mer) in complex with p17 dimer (represented as electrostatic surface). Zoom insets: interactions before and after MDs are reported. P17 amino acids and monosaccharides involved in the networks are in sticks, blue and yellow.

The sliding window and incremental docking methods were used to identify and join the best binding poses of each 4-mer heparin in the traced paths (Fig. 5c), predicting two heparin chain models: the 15-mer heparin, modelled on the shorter path, and the 24-mer heparin modelled on the longer path (Fig. 6).

In the complexes formed by p17 dimer with the 15- and 24-mer heparins, H-bonds are mainly formed with SO_3_^−^ or COO^−^. In the 15-mer heparin complex H-bonds occur mainly within the N-HBD domains and α-helix 2 of both monomers (Fig. 6a and Table S1), while in the 24-mer heparin complex they occur in all the helices of both monomers and in the C-HBDs (Fig. 6b and Table S2).

MDs were performed to evaluate the stability, conformational drift and refine the two heparin/p17 dimer complexes. Root mean square fluctuation (RMSF) analysis of the 15-mer heparin/p17 dimer complex shows that the C-termini of both monomers are the most variable regions (fluctuation up to 5 Å), in agreement with relaxation and amide proton exchange studies^27^. Three other flexible regions span from 7–14, 20–30 and 60–75 (Fig. S3a). The 15-mer heparin backbone is stable, while its SO_3_^−^ are endowed with a higher fluctuation due to solvent exposure. In particular, monosaccharides from 10 to 14 exhibit the highest fluctuation (up to 5 Å), due to their interaction with N-HBD of p17 monomer B (Fig. S3b).

Root mean square deviation (RMSD) of the p17 C-α globular domain is stable during the simulations (average fluctuation = 0.5 Å) while the C-termini of both the monomers, due to solvent exposure, reach stability only after 25 and 45 ns. Heparin remains stable during the simulation (average fluctuation = 2 Å). It also reaches a second stability plateau after 28 ns due to the fitting upon binding of the 8^th^ monosaccharide (Fig. S3c).

Electrostatic surface analysis of docking model shows that 15-mer heparin sets up an articulate network of interactions inside the cationic N-HBDs of monomer A and B, between which is interposed a negative electrostatic surface potential generated by the exposure of COO^−^ of E_40_, by the N-SO_3_^−^ and 3-OH^−^ of the 10^th^ and 8^th^ Glc which are engaged in H-bonds with R_39_ and R_43_ of α-helix 2 of monomer B (Fig. 6a and Table S1). However, MDs show that, after 20 ns, these two H-bonds are lost due to solvent exposure of N-SO_3_^−^ and 3-OH^−^, while a new H-bond network is formed between the COO^−^ of E_40_ and the guanidinium groups of R_39_ and R_43_ and between the N-SO_3_^−^ of the 10^th^ Glc and the guanidinium group of R_39_ (Fig. 6a and Table S1). This new arrangement induced by the heparin conformational drift leads to the formation of a continuous positive channel in the p17 dimer structure (Fig. 6a).

H-bond analysis shows that the 15-mer heparin makes an articulate network of H-bonds with the p17 dimer (Table S1). Worth of mention, K_30_ interacts with 2-O-SO_3_^−^ and 3-OH^_^ of the 3^rd^ IdoA and with 6-O-SO_3_^−^ of the 4^th^ Glc with an average persistency of 65%, while H_33_ interacts with 6-O-SO_3_^−^ of the 4^th^ Glc with an average persistency of 50%. Interestingly, the H-bond persistency of the last two residues together is higher than 80%, indicating a synergic effect mainly due to the groups of the the 3^rd^ IdoA. Furthermore, R_39_ and R_43_ of monomer B interact simultaneously with E_40_ and heparin (persistency = 50% for both), further stabilizing the interaction network between the protein and the sugar.

RMSF analysis of the 24-mer heparin/p17 dimer complex shows that the C-terminus of p17 monomer B displays a lower fluctuation in respect to that in the 15-mer heparin/p17 dimer complex, due to a better H-bond network and a lower exposure to solvent, in particular in the second half of the heparin chain (monomers 12–24) (Fig. S3d and Table S2). As already highlighted in the 15-mer heparin/p17 dimer complex (Fig. S3e), a higher fluctuation can be instead appreciated for monomer A binding to the first half of the heparin chain (monomers 1–12) for which a greater fitting needs to be induced to generate the positive channel in the protein. More into the details, the first four monosaccharides of heparin, that interact with the N-terminus of monomer A, are endowed with the higher fluctuation, with their SO_3_^−^ showing an average fluctuation up to 2 Å, likely due to their solvation.

RMSD analysis demonstrates that the p17 C-α globular domains in the dimers are stable during the simulation, with an average fluctuation = 1 Å. This stability is shared also by the C-terminus of monomer B, while the C-terminus of monomer A and heparin reach stability only after 35 ns (Fig. S3f).

Electrostatic analysis of docking model shows that some SO_3_^−^ of 24-mer heparin take contact with the N-ter basic motif of both the p17 monomers and with the C-ter of monomer B (Fig. 6b and Table S2) while some other remain instead exposed to the solvent. However, MDs shows that these free SO_3_^−^, by inducing molecular drifts, succeed in taking contact with basic residues of the C-HBDs of both the p17 monomers. Thus, the heparin-induced conformational drift of p17 monomers creates sandwich-like structures that lead to the formation of the continuous positive channel, already described in literature^27^, that prevents solvent exposition of bound heparin (Fig. 6b and Table S2). The results of the H-bonds analysis of the 24-mer heparin/p17 dimer complex are reported in Table S2. Considering the whole simulation, residues A_115_ and Q_116_ of the C-terminus of monomer A, that exhibit low H-bond persistency with heparin (55 and 29%), increase their persistency after 35 ns to 99% and 85%, due to binding-induced stabilization of both heparin and p17 C-termini.

In conclusion, the new computational protocol here adopted discloses the possibility that heparin chains can connect two p17 monomers with different binding modes depending on their lengths and that, during interaction, heparin induces dynamic conformational drift of the p17 dimer that facilitate the interaction between the GAG and the protein monomers, stabilizing the dimer and likely promoting further oligomerization.

### Heparan sulfate-dependent p17 oligomerization at the lymphoblastoid cell surface

**Figure 7:**
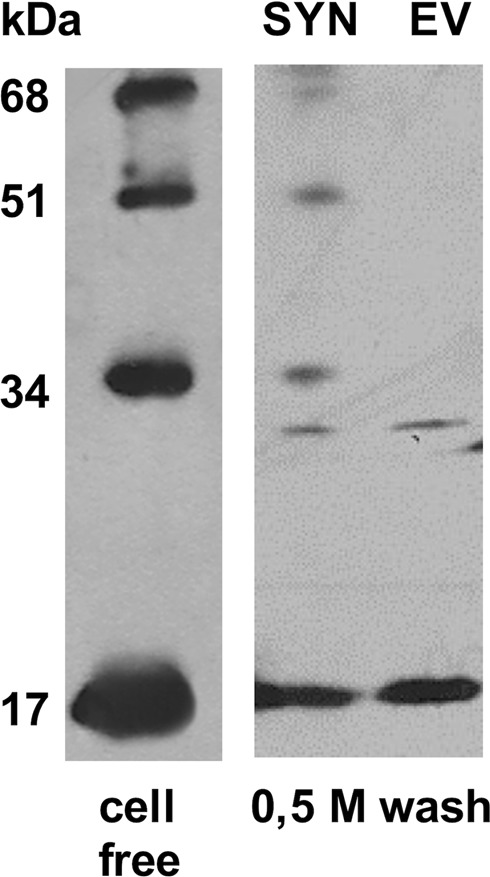
HSPGs-dependent p17 oligomerization at the surface of lymphoblastoid cells. Namalwa cells devoid of HSPGs (EV) or overexpressing the HSPG Syndecan-1 (SYN) were incubated with p17 and subjected to cross-link. Then, p17 associated to the cell surface (1.0 M wash) was analyzed in WB. A representative blot from a p17 oligomerization experiment performed with the purified protein (cell free) is reported for comparison.

Heparin, that is produced and released by mast cells in the blood, is generally used experimentally as a structural analogue of HSPGs expressed on the surface of almost all eukaryotic cells, were they act as receptors for many proteins. Thus, once characterized the capacity of heparin to promote p17 oligomerization at a biochemical and computational level, we wondered if this capacity was shared by HSPGs associated to lymphoblastoid cells. We thus used the Namalwa cell model already exploited to study the HSPGs with HIV-1 Tat/HSPGs interaction^12^: p17 effectively oligomerizes at the surface of SYN-Ncs, originating those same high orders oligomers characterized by WB in the cell-free system. p17 oligomerization at the cell surface specifically depends on Syndecan-1, since it does not occur on HSPGs-deficient EV-Ncs (Fig. 7). Interestingly, an additional 30 kDa band is visible in both SYN- and EV-Ncs, possibly representative of p17 interaction with an unknown protein present at the surface of both clones.

**Figure 8:**
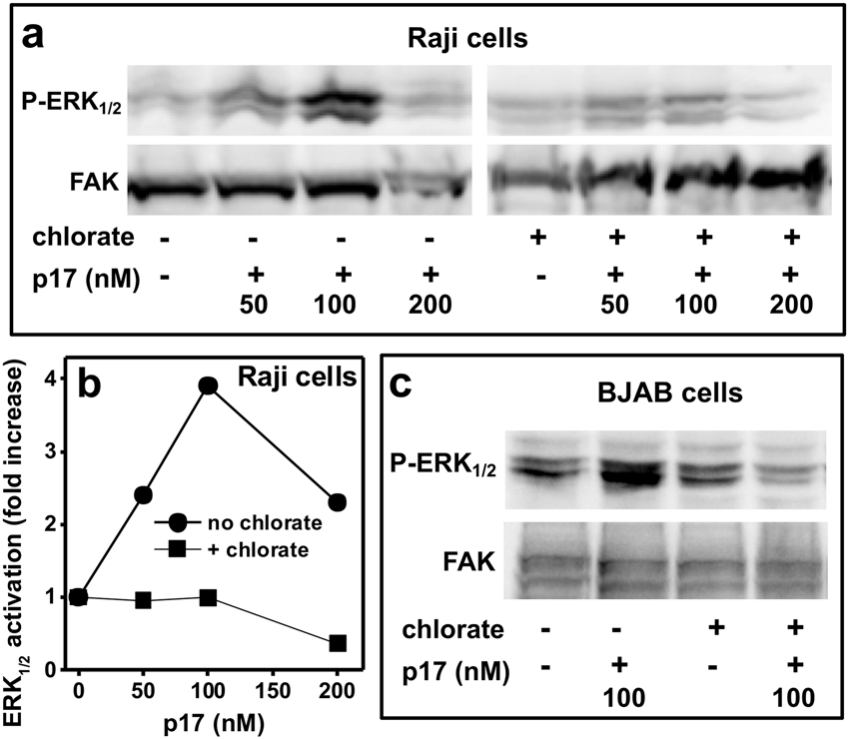
Role of HSPGs in p17-induced ERK_1/2_ activation in lymphoblastoid cells. Raji (**a,b)** or BJAB **(c)** cells were left untreated or treated with chlorate and incubated with the indicated amount of p17. Then, ERK_1/2_ activation was evaluated. For both panel a and c, the experiment shown is representative of another one that gave similar results.

We finally investigated if p17 binding to HSPGs and the consequent p17 oligomerization at the lymphoblastoid cell surface were functionally relevant. Since SO_3_^−^ of heparin are required for its p17-oligomerizing capacity, we treated Raji and BJAB cells with chlorate, that inhibits sulfation of HS and that has been already used to study HSPGs functions in lymphoid cells^12^. Chlorate-treated cells were evaluated for their sensitivity to p17 stimulation by evaluating extracellular-regulated kinase_1/2_ (ERK_1/2_) phosphorylation, a second messenger involved in p17-dependent lymphoid cell activation^6,17^: p17 induces a dose-dependent, bell-shaped activation of ERK_1/2_ in Raji cells that is abolished by chlorate-treatment (Fig. 8a,b). The same results were obtained with BJAB cells (Fig. 8c). Taken together, these results indicate that, at the surface of lymphoblastoid cells, the binding of p17 to HSPGs and possibly the consequent p17 oligomerization are required to activate signal transduction.

## Discussion

Protein oligomerization is involved in a variety of biological processes. It can occur in the form of “simple” dimers or higher order oligomers of cytokines, chemokines and growth factors that favour receptor clustering at the cell surface and transduction of the signal that, in turn, trigger cytoskeletal rearrangements, cell movement and other cellular responses including proliferation and survival involved in physiological and pathological processes such as immune responses, angiogenesis and cancerogenesis^14,28–31^. Differently, some “amyloidogenic” proteins are endowed with the propensity to change conformation from an α-helix to β-sheet, tending to misfold and to form aspecific large aggregates responsible of the pathogenesis of many human diseases, including neurodegenerative pathologies such as Alzheimer and Parkinson’ disease^32^.

p17 undergoes trimerization during viral assembly in the proximity of the inner cell surface^33^, where it reaches the millimolar range of concentration^34^. Compatible with this high concentration, here we found that p17 spontaneously self-interacts with a relatively low affinity (*Kd* ≅ 600 nM). p17 oligomerization is prevented by DTT and urea, indicating that this process requires a proper 3D conformation of p17 and suggesting that, in our experimental conditions, it is correctly folded. Accordingly, circular dichroism spectroscopy revealed that our recombinant p17 displays a dominant α-helical structure in aqueous solution^35^, consistent with the known structural features of p17^20^.

p17 is released by HIV-1-infected cells in the extracellular environment, where it acts as a cytokine, with its bioavailability and activity regulated by heparin or HSPGs^10^. At variance with the high p17 concentrations that can be found inside HIV-infected cells, released p17 can be found in body fluids at the nanomolar range of concentrations^3,4^, suggesting that, in this environment it likely does not oligomerize. Importantly, extracellular protein oligomerization of many physiological or pathological cytokines is often regulated by free heparin and cell-surface HSPGs^13,14,28^.

Here, by exploiting chemical cross-linking, already used to study FGF2 oligomerization^36^, we found that, beside the already known trimer, free p17 spontaneously forms dimers and tetramers and that p17 oligomerization is promoted by heparin in a ionic strength-dependent way, with 1.0 M NaCl necessary to achieve a complete complex dissociation. Interestingly, the spontaneous p17 trimers are instead almost completely disrupted by 0.5 M NaCl^17^, suggesting that heparin not only increases p17 oligomerization, but stabilizes it by increasing its affinity, making it possible to occur also at the cell surface and in the extracellular environment, where p17 concentrations are significantly lower^3,4^.

Heparin-induced p17 oligomerization occurs only in the presence of low GAG concentrations. It is tentative to hypothesize that a molar excess of p17 favors its multiple binding to adjacent sites on a single heparin chain. Differently, in the presence of higher concentrations of heparin, the large availability of binding sites on separate GAG chains in the presence of a limited amount of protein likely lowers the chances of p17 molecules to be close enough to self-interact. Interestingly, a similar behavior was already observed for heparin-induced FGF2 dimerization^37^.

As already mentioned in introduction, the C-terminal portion^19^ and amino acids E_42_-N_47_, Q_59_, Q_63_, and Q_69_-E_74_^20^ of p17 mediate its oligomerization. Our MDs predict a heparin-induced drift of p17 monomers that leads to the resetting of globular domains and loss of interaction only between A_45_ of monomer A and S_72_ of monomer B that, in turn, induces the alignment of both the α-helix 2. Thus, heparin could bring together multiple copies of p17 through the relatively high affinity p17/heparin interaction (*Kd* ≅100 nM)^10^, leaving exposed the p17 self-assembly regions on adjacent proteins as to facilitate the low affinity p17/p17 interaction (*Kd* ≅ 600 nM). Also, it is possible that the formation of high order oligomers (trimer and tetramers) can be triggered by the initial assembly of the p17 dimer induced by heparin. In effect, oligomerization is often the outcome of a cooperative interaction process, during which a first binding makes easier the following ones^13^. Thus, the action of heparin may consist in providing the appropriate scaffold to facilitate and stabilize the relatively weak p17/p17 interaction, as already observed for different other factors and cytokines, including FGF2^38^, HGF^39^ and platelet-derived growth factor^40^.

Beside heparin, also HSPGs of lymphoblastoid cell surface bind p17 favoring its oligomerization and are required to induce p17-dependent ERK_1/2_ phosphorylation, suggesting the possibility that, *in vivo*, p17 binding to HSPG and the following oligomerization at the cell surface are required for a full lymphoid cell activation, concurring to pathological effects exerted by p17 during AIDS progression.

The capacity of heparin to promote the formation of p17 tetramers and even examers^18^ infers the possibility that it could favor the formation of even higher order p17 aggregates. In effect, after prolonged exposures, in our WB analyses very high molecular weight bands are detectable both in solution and at the cell surface (data not shown). On the other hand, at very high concentration (4.0 μM, that is 40 times higher than that used in our experimental conditions) p17 generates amyloidogenic assemblies^4^. Thus, it is tentative to hypothesize that, at low concentrations and with the help of heparin/HSPGs, p17 forms specific oligomers that are functional to the activation of specific p17 receptors and hence to the cytokine-like activity of the protein. At variance, in peculiar sites of accumulation, p17 reaches high concentration undergoing misfolding and aspecific aggregation that contribute to AIDS-associated neurodegeneration^4^. We can not rule out however the possibility that heparin plays a role also in p17 amyloidogenic oligomerization, as already demonstrated for other GAGs in various amyloid diseases^41^. To properly investigate this possibility, suitable techniques such as analytical ultra-centrifugation approach or atomic force and transmission electron microscopy^4^ should be employed.

As mentioned in the introduction, computational models for heparin/protein interaction have been so far restricted to docking studies with short GAG chains and aimed at the identification of HBDs in proteins. Here, we have set up a new “step by step” protocol (Fig. S2) by which it has been possible to identify two heparin paths and then create two computational models of a p17 dimer in complex with 15- and 24-mer heparins whose validity is confirmed by their good agreement with the experimental data. Indeed, docking and MDs predict 15-mer and 24-mer heparin to bind to p17 dimer through an articulate network of interactions occurring mainly between the negative SO_3_^−^ of heparin and the positive amino acids of the proteins. Accordingly, these interactions have been experimentally demonstrated to mediate heparin/p17 interaction and p17 oligomerization by experiments with NaCl, desulfated heparin and the p17 K → A mutant.

MDs studies with the heparin chains models disclosed the capacity of heparin to interact with a p17 dimer with different binding modes depending on its length. Also, heparin can induce a “fitting-upon-binding” on the p17 dimer that causes the exposition of positively charged residues on the globular domains and the warping of both the C-termini to create a sandwich-like structure around heparin. Finally, RMSF analysis showed that when complexed with the 24-mer heparin, the p17 dimer complex displays a lower fluctuation in respect to when complexed with 15-mer heparin, suggesting that the longer is the heparin chain, the stronger is its stabilizing effect on the p17 dimer. MDs also show that, during the binding process, heparin fits together with the protein, simulating an annular-like structure already described in literature^4^.

Beside p17, this computational protocol could be useful to understand at an atomic level the role of heparin in the processes of oligomerization of a variety of cytokines, chemokines and growth factors, and even in the formation of ternary complexes among heparin, heparin-binding proteins and their receptors.

## Material and Methods

### Computational studies

#### Models

p17 and the heparin tetrasaccharide (4-mer heparin) were modelled as described^18^. A 4-mer heparin probe with the deletion of the H atom of the hydroxyl groups at position 4 in the first IdoA and of the hydroxyl group at position 1 of the Glc (4-mer heparin modified probe) was prepared and used in docking simulations to promote the 1 → 4 glycosidic linkage. Two p17 mutants were modelled by Alanine Scanning Method in which the lysine residues of both the N- and C-terminal basic domains were replaced with alanine (N-ter K → A p17 and NC-ter K → A p17).

#### Heparin path identification

Blind docking simulations were performed by ClusPro web-server^42^ using 4-mer heparin and N-ter K → A or NC-ter K → A p17 dimers to identify heparin-binding regions on p17 other than those in the N- and C-ter basic domains. The identified heparin probe sites were then filtered by best score, cluster size, visual inspection and positioned in a dimer of wild type (wt) p17 to finally achieve an alignment and hence a traced heparin path.

#### Incremental docking and heparin modelling

The 4-mer heparin modified probe was used in local docking simulation along the traced heparin path in the wt p17 dimer by Autodock 4.2^43^. The “sliding window method” was set up to create a sequence of overlapping sliding grids, each covering a whole 4-mer heparin and the last saccharide unit of the previous one. Local docking poses were filtered for free energy of binding, clusters size and correct orientation. The aligned 4-mer heparin modified probes were joined by 1 → 4 glycosidic linkages using Pymol^44^. Gasteiger-Hückel charges were assigned to the sugar and then minimized using steepest descendent and conjugate gradient methods by Chimera^45^, obtaining heparin chains of increasing length.

#### Molecular dynamic simulation (MDs)

Amber14 package^46^ was used for MDs of p17 dimer in complex with 15- and 24-mer heparins. MDs were carried out using ff99SB force field parameters for protein and GLYCAMo6 for heparins. Each complex was neutralized by adding Ca^2+^ restrained away from the protein^47^ and solvated with TIP3P water model.

Energy minimization was carried out with the non-bonded cut-off of 8 Å through the following steps: (i) p17/heparin complexes and counter ions were restrained by a harmonic potential of 5 kcal/mol × Å^2^, while water molecules were relaxed using 2,500 cycles of steepest descent and conjugate gradient methods; (ii) counter ions and hydrogens were relaxed using 5,000 cycles of steepest descent and conjugate gradient methods and restrained by a harmonic potentials of 3 kcal/mol × Å^2^ and then of 1 kcal/mol × Å^2^; (iii) the system was relaxed using 5,000 cycles of steepest descent and conjugate gradient methods without any restraint; iv) the system was heated from 0.1 K to 100.0 K in NVT (constant volume) and from 100.0 to 300.0 K in NPT (1.0 atm constant pressure). The two generated complexes were simulated in a periodic boundary conditions using the Langevin algorithm at 300.0 K. During heating and simulations the Ca^2+^ ions were restrained at 500 kcal/mol × Å^2^.

Equilibration (3 ns) and simulation were validated using the physical observables parameters of the system confirming that the complexes obeyed the NPT ensemble. Electrostatic interactions were calculated using the Particle Mesh Ewald method. A cut-off of 8.0 Å was applied to van der Waals forces. Integration time step was set to 1.0 fs during equilibration and 2.0 fs during simulation. MDs were performed over 50 ns using the pmemd CUDA program of the Amber14 package and a server Tesla K20 Graphical Processing Unit.

### Reagents

wt HIV-1 matrix protein p17, its N-ter K → A mutant, obtained by the substitution of K to A residues in the N-HBD (Fig. S1) and anti-p17 antibody MBS-3 were produced and purified as described^17^. Chemical cross-linker bis(sulfosuccinimidyl)suberate (BS3) was from ThermoFisher Scientific (Waltham, MA). Anti-focal adhesion kinase (FAK, sc-557) antibody and anti P-ERK_1/2_ from Santa Cruz Biotechnology (Santa Cruz, CA) and Cell Signaling Technology (Danvers, MA), respectively. Peroxidase-coupled goat anti-mouse and anti-rabbit IgG from Sigma (St Louis, MO) and Biorad (Hercules, CA), respectively. Conventional unmodified heparin (13.6 kDa) from Laboratori Derivati Organici S.p.A., Milan, Italy. Selectively desulfated heparins were a gift of Ronzoni Institute, Milan^48^.

### Surface plasmon resonance (SPR) binding assay

SPR measurements were performed on a BIAcore X 100 (GE-Healthcare, Milwaukee, WI). p17 (10 μg/ml in sodium acetate buffer 10 mM pH 4.8) was immobilized onto one of the two flow cells of a CM5 sensorchip [2,500 resonance units (RU), ≅ 0.15 fmoles/mm2] by amine-coupling method as for manufacturer’s instructions. The second flow cell was left void and used for blank subtraction. For binding analysis, increasing concentrations of p17 in 10 mM HEPES pH 7.4, 150 mM NaCl, 3 mM EDTA, and 0.005% surfactant P20 (HBS-EP) were injected over the p17- or void surface for 2 min. and then washed until dissociation. After each run, the sensorchip was regenerated by injection of HBS-EP containing 2.0 M NaCl. Association and dissociation rates (*kon* and *koff*) and dissociation constant (*Kd*) were calculated from blank-subtracted sensorgrams overlay using the non linear fitting (single site model) software package BIAevaluation 3.2. Also, *Kd* was calculated by being fitted with the proper form of Scatchard’s equation for the plot of the bound RU at equilibrium *versus* the ligand concentration in solution using two different softwares (Prism GraphPad and Origin Microcal).

### p17 oligomerization Western blot (WB) assay

wt or N-ter K → A p17 (2 μg/ml in 20 μl) were incubated for 2 h at 37 °C in PBS alone or with Urea, DTT, unmodified or selectively desulfated heparins or NaCl. Protein oligomers were chemically cross-linked by a 2 h incubation at 37 °C with 1 mM BS3. Then, samples were incubated for 5 min. at 90 °C in reducing sample buffer and run onto a SDS 12% PAGE. Proteins were transferred onto a PVDF membrane (Millipore, Bedford, MA) that was then incubated for 18 h at 4 °C with anti-p17 antibody [1:1000 in Tris-HCl 10 mM pH 7.5, NaCl 150 mM, 0.1% TWEEN 20 (TTBS)]. Antigen-antibody complexes were detected by a 1 h incubation at 4 °C with peroxidase-coupled goat anti-mouse IgG (1:5000 in TTBS) and the ECL System (Santa Cruz Biotechnology). The 17, 34, 51 and 68 kDa bands, corresponding to p17 monomer and higher-order oligomers, were quantified by computerized image analysis using Image LabTM software (Bio-Rad).

### Cell cultures

Burkitt lymphoma-derived Namalwa cell clones genetically deficient for HSPGs expression were transfected with the empty vector or with a vector containing syndecan-1 cDNA (EV-Ncs and SYN-Ncs) and cultured as described^49^. BJAB and Raji human B-lymphoblastoid cells were from American Type Culture Collection and were cultured in RPMI-1640 medium containing 10% fetal calf serum (FCS), 100 U/mL penicillin, 50 mg/ml streptomycin, 1 mM L-glutamine.

### Oligomerization of p17 at the surface of lymphoblastoid cells

Aliquots (1,5 × 10^6^ cells) of EV- or SYN-Ncs were incubated for 2 h at 37 °C in culture medium with p17 (1 μg/ml) without serum. Then, chemical cross-linker BS3 (1 mM) was added and incubated for 30 min. at room temperature. At the end of incubation, cells were washed with PBS to remove unbound p17 and with 0.5 M NaCl in PBS to remove proteins weakly adsorbed to the cell surface. Finally, cells were washed with 1.0 M NaCl in PBS to detach HSPG-associated proteins. Aliquots of the 1.0 M wash (40 μl) were analyzed in WB as described above.

### ERK_1/2_ activation assay

Raji or BJAB cells were incubated for 72 h in RPMI-1640 medium containing 10% FCS with or without 45 mM NaClO_3_ (chlorate) and resuspended in serum-free medium. Aliquots (1,5 × 10^6^ cells) were then further incubated for 30 min. with serum-free medium with increasing concentrations of p17. At the end of the incubation, cells were lysed in 50 mM Tris-HCl pH 7.4 containing 150 mM NaCl, 1% Triton X-100, 0.1% Brij and Protease Inhibitor Cocktail (Sigma) and centrifuged. Pellets were resuspended in reducing SDS-PAGE sample buffer, incubated for 5 min. at 90 °C and analyzed on SDS-12% PAGE followed by WB using anti-P-ERK_1/2_ antibodies. Equal loading of the lanes was confirmed by immunoblotting with anti-FAK antibodies. The intensity of the ERK_1/2_ signal was quantified as described above and normalized to the intensity of the corresponding FAK band.

## Supplementary information

supplementary figures and tables

original blots

## Data Availability

Materials, data and protocols will be made available to readers from the corresponding author on reasonable request.

## References

[1] Bryant M, Ratner L (1990). Myristoylation-dependent replication and assembly of human immunodeficiency virus 1. Proc Natl Acad Sci USA.

[2] Cannon PM (1997). Structure-function studies of the human immunodeficiency virus type 1 matrix protein, p17. J Virol.

[3] Fiorentini S, Giagulli C, Caccuri F, Magiera AK, Caruso A (2010). HIV-1 matrix protein p17: a candidate antigen for therapeutic vaccines against AIDS. Pharmacology & therapeutics.

[4] Zeinolabediny Y (2017). HIV-1 matrix protein p17 misfolding forms toxic amyloidogenic assemblies that induce neurocognitive disorders. Scientific reports.

[5] Li S, Bozzo L, Wu Z, Lu W, Romerio F (2010). The HIV-1 matrix protein p17 activates the transcription factors c-Myc and CREB in human B cells. The new microbiologica.

[6] Martorelli D (2015). A natural HIV p17 protein variant up-regulates the LMP-1 EBV oncoprotein and promotes the growth of EBV-infected B-lymphocytes: implications for EBV-driven lymphomagenesis in the HIV setting. International journal of cancer.

[7] De Francesco MA, Poiesi C, Ricotta D, Manca N (2006). HIV p17 reverses the anti-inflammatory activity of IL-4 on IL-15 stimulated monocytes and modulates their ability to secrete MIP-1 alpha. Virus Res.

[8] De Francesco MA (2002). HIV-1 matrix protein p17 increases the production of proinflammatory cytokines and counteracts IL-4 activity by binding to a cellular receptor. Proceedings of the National Academy of Sciences of the United States of America.

[9] Rusnati M, Oreste P, Zoppetti G, Presta M (2005). Biotechnological engineering of heparin/heparan sulphate: a novel area of multi-target drug discovery. Current pharmaceutical design.

[10] Bugatti A (2013). Molecular interaction studies of HIV-1 matrix protein p17 and heparin: identification of the heparin-binding motif of p17 as a target for the development of multitarget antagonists. The Journal of biological chemistry.

[11] Roderiquez G (1995). Mediation of human immunodeficiency virus type 1 binding by interaction of cell surface heparan sulfate proteoglycans with the V3 region of envelope gp120-gp41. J Virol.

[12] Urbinati C (2009). HIV-1 Tat and heparan sulfate proteoglycan interaction: a novel mechanism of lymphocyte adhesion and migration across the endothelium. Blood.

[13] Rusnati M (1999). Multiple interactions of HIV-I Tat protein with size-defined heparin oligosaccharides. The Journal of biological chemistry.

[14] Hoogewerf AJ (1997). Glycosaminoglycans mediate cell surface oligomerization of chemokines. Biochemistry.

[15] Alfadhli A, Still A, Barklis E (2009). Analysis of Human Immunodeficiency Virus Type 1 Matrix Binding to Membranes and Nucleic Acids. Journal of virology.

[16] Saad JS (2006). Structural basis for targeting HIV-1 Gag proteins to the plasma membrane for virus assembly. Proceedings of the National Academy of Sciences of the United States of America.

[17] Giagulli C (2011). Opposite effects of HIV-1 p17 variants on PTEN activation and cell growth in B cells. PloS one.

[18] De Matteis MA, Godi A (2004). PI-loting membrane traffic. Nature cell biology.

[19] Verli H, Calazans A, Brindeiro R, Tanuri A, Guimaraes JA (2007). Molecular dynamics analysis of HIV-1 matrix protein: clarifying differences between crystallographic and solution structures. Journal of molecular graphics & modelling.

[20] Hill CP, Worthylake D, Bancroft DP, Christensen AM, Sundquist WI (1996). Crystal structures of the trimeric human immunodeficiency virus type 1 matrix protein: implications for membrane association and assembly. Proceedings of the National Academy of Sciences of the United States of America.

[21] Doherty, R. S. *et al*. BioAfrica’s HIV-1 Proteomics Resource: Combining protein data with bioinformatics tools. *Retrovirology* **2** (2005). 10.1186/1742-4690-2-18 PMC555852 15757512

[22] Mottarella SE (2014). Docking server for the identification of heparin binding sites on proteins. Journal of chemical information and modeling.

[23] Mulloy B, Forster MJ, Jones C, Davies DB (1993). N.m.r. and molecular-modelling studies of the solution conformation of heparin. The Biochemical journal.

[24] Babik S, Samsonov SA, Pisabarro MT (2017). Computational drill down on FGF1-heparin interactions through methodological evaluation. Glycoconjugate journal.

[25] Uciechowska-Kaczmarzyk U (2018). Molecular dynamics-based model of VEGF-A and its heparin interactions. Journal of molecular graphics & modelling.

[26] Brown, A. J., Sepuru, K. M. & Rajarathnam, K. Structural Basis of Native CXCL7 Monomer Binding to CXCR2 Receptor N-Domain and Glycosaminoglycan Heparin. *International journal of molecular sciences* **18** (2017). 10.3390/ijms18030508 PMC5372524 28245630

[27] Ohori Y (2014). Flexible and rigid structures in HIV-1 p17 matrix protein monitored by relaxation and amide proton exchange with NMR. Biochimica et biophysica acta.

[28] Proudfoot AE (2003). Glycosaminoglycan binding and oligomerization are essential for the *in vivo* activity of certain chemokines. Proceedings of the National Academy of Sciences of the United States of America.

[29] Wang X, Sharp JS, Handel TM, Prestegard JH (2013). Chemokine oligomerization in cell signaling and migration. Progress in molecular biology and translational science.

[30] Chiodelli P, Bugatti A, Urbinati C, Rusnati M (2015). Heparin/Heparan sulfate proteoglycans glycomic interactome in angiogenesis: biological implications and therapeutical use. Molecules.

[31] Rusnati M, Presta M (2015). Angiogenic growth factors interactome and drug discovery: The contribution of surface plasmon resonance. Cytokine & growth factor reviews.

[32] Chiti F, Dobson CM (2017). Protein Misfolding, Amyloid Formation, and Human Disease: A Summary of Progress Over the Last Decade. Annual review of biochemistry.

[33] Rusnati M, Chiodelli P, Bugatti A, Urbinati C (2015). Bridging the past and the future of virology: surface plasmon resonance as a powerful tool to investigate virus/host interactions. Critical reviews in microbiology.

[34] Massiah MA (1996). Comparison of the NMR and X-ray structures of the HIV-1 matrix protein: evidence for conformational changes during viral assembly. Protein science: a publication of the Protein. Society.

[35] Giagulli C (2017). A single amino acid substitution confers B-cell clonogenic activity to the HIV-1 matrix protein p17. Scientific reports.

[36] Platonova N (2014). Dimerization capacities of FGF2 purified with or without heparin-affinity chromatography. PloS one.

[37] Casu B (2002). Short heparin sequences spaced by glycol-split uronate residues are antagonists of fibroblast growth factor 2 and angiogenesis inhibitors. Biochemistry.

[38] Ornitz DM (1992). Heparin is required for cell-free binding of basic fibroblast growth factor to a soluble receptor and for mitogenesis in whole cells. Molecular and cellular biology.

[39] Stahl SJ (1997). Functional and biophysical characterization of recombinant human hepatocyte growth factor isoforms produced in Escherichia coli. The Biochemical journal.

[40] Stringer SE, Gallagher JT (1997). Specific binding of the chemokine platelet factor 4 to heparan sulfate. The Journal of biological chemistry.

[41] Lindahl U, Kjellen L (2013). Pathophysiology of heparan sulphate: many diseases, few drugs. Journal of internal medicine.

[42] Kozakov D (2017). The ClusPro web server for protein-protein docking. Nature protocols.

[43] Morris GM (2009). AutoDock4 and AutoDockTools4: Automated docking with selective receptor flexibility. Journal of computational chemistry.

[44] DeLano WL (2002). Unraveling hot spots in binding interfaces: progress and challenges. Current opinion in structural biology.

[45] Pettersen EF (2004). UCSF Chimera–a visualization system for exploratory research and analysis. Journal of computational chemistry.

[46] Yang L (2006). New-generation amber united-atom force field. The journal of physical chemistry. B.

[47] Drabik P, Liwo A, Czaplewski C, Ciarkowski J (2001). The investigation of the effects of counterions in protein dynamics simulations. Protein engineering.

[48] Inoue Y, Nagasawa K (1976). Selective N-desulfation of heparin with dimethyl sulfoxide containing water or methanol. Carbohydr Res.

[49] Zhang Z, Coomans C, David G (2001). Membrane heparan sulfate proteoglycan-supported FGF2-FGFR1 signaling: evidence in support of the “cooperative end structures” model. J Biol Chem.

