# Supplementary material for: Heparin and heparan sulfate proteoglycans promote HIV-1 p17 matrix protein oligomerization: computational, biochemical and biological implications
Source: Sci Rep. 2019 Oct 31;9:15768. doi: 10.1038/s41598-019-52201-w (PMC6823450; doi:10.1038/s41598-019-52201-w)
Supplement: Supplementary file 1 — supplementary figures and tables [file 41598_2019_52201_MOESM1_ESM.pdf]

Heparin and heparan sulfate proteoglycans promotes HIV-1 p17 matrix protein oligomerization: computational, biochemical and biological implications.

Antonella Bugatti, Giulia Paiardi, Chiara Urbinati, Paola Chiodelli, Alessandro Orro, Matteo Uggeri, Luciano Milanesi, Arnaldo Caruso, Francesca Caccuri, Pasqualina D’Ursi and Marco Rusnati

SUPPLEMENTARY TABLES and FIGURES

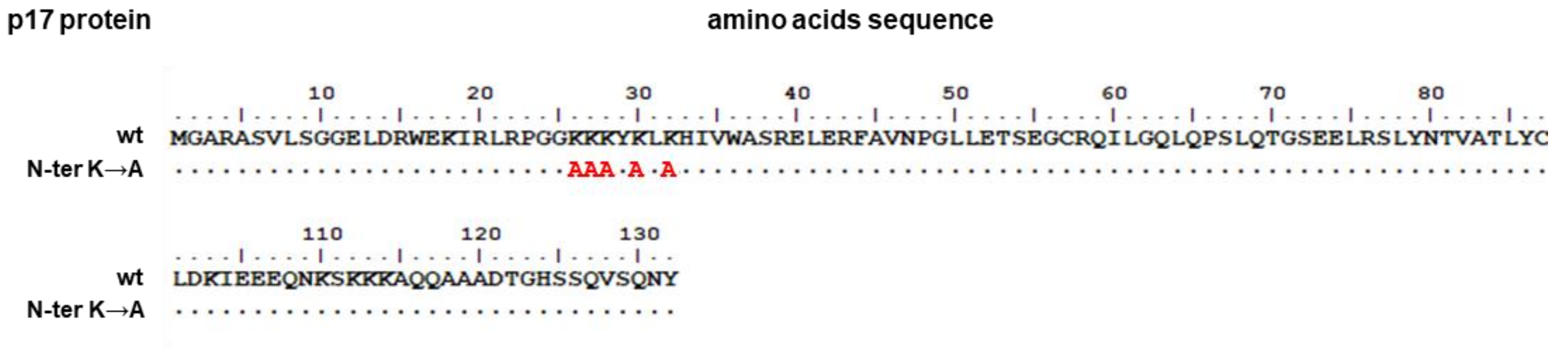

Suppl. Fig. S1: amino acid sequences of wt HIV-1 matrix protein p17 and its N-ter K→A mutant

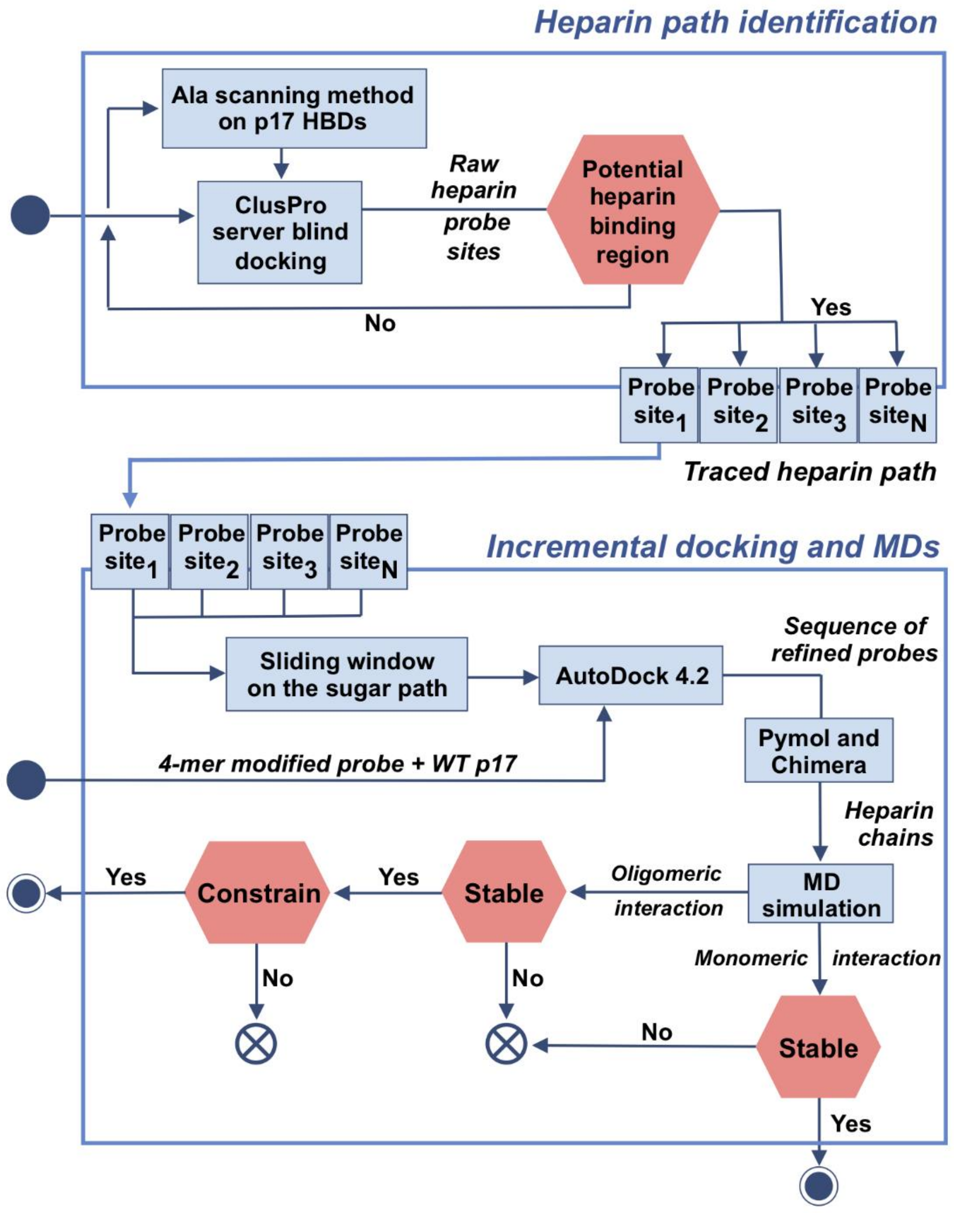

Suppl. Fig. S2: Workflow of the computational studies

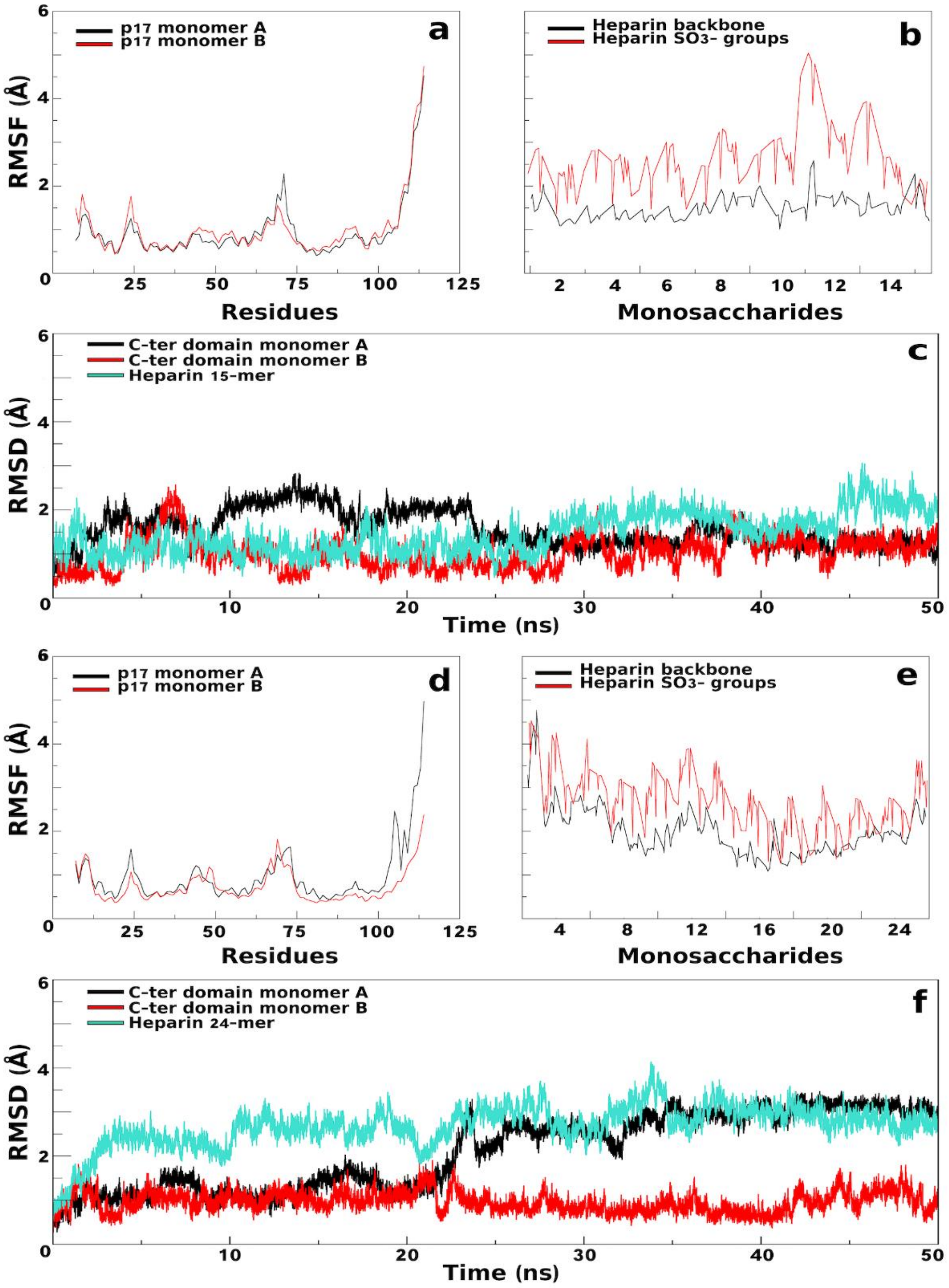

Suppl. Fig. S3: RMS analyses. Analysis of RMSD and RMSF from MDs trajectories were performed to evaluate the stability and conformational drift of the complexes. RMSD was used to evaluate the stability of unbound p17 (considering its globular domain and C-terminal region separately), and heparin (considering its backbone and sulphate groups separately) while RMSF were calculated for single p17 amino acids or for heparin atoms. Starting from MDs trajectories and using CPPTRAJ, the most representative cluster and H-bond were assessed for p17/heparin interactions and stability. In figure are reported RMSF profile of p17 dimer in complex with 15- **(a)** or 24- **(d)** and of 15-mer **(b)** or 24-mer **(e)** heparin calculated per atoms and displayed per monosaccharides. RMSD along the simulation are also reported for 15-mer **(c)** or 24-mer **(f)** heparin in complex with the p17 dimer.

SUPPLEMENTARY TABLES

Suppl. Tab. 1: H-bond analysis in the 15-mer heparin/p17 dimer complex from docking or MDs. The 15-mer heparin makes stable contact with amino-lysine and guanidine-arginine groups of the residues R_{22} (87%), K_{26} (99%), K_{27} (99%), K_{32} (95%), R_{39} (97%), R_{43} (93%) of p17 monomer A and with K_{26} (98%), K_{27} (98%), K_{28} (87%), K_{30} (86%), K_{32} (92%) of p17 monomer B. In brackets are the average persistency of the different H-bonds. P17 amino acids in loop 1 are in red, those in the N-terminal HBD in blue and those in the α-helix 2 in green. IdoA: 2-O-sulfated L-iduronic acid. Glc: N, 6-O-disulfate D-glucosamine. Hc: heterocyclic. n.d.: not defined. *H-bond with heparin is mediated by the N atom of the peptide bond. In all the other cases, N atoms of the side chain is instead involved.

		**docking**		**MDs**	
**heparin unit**	**p17 monomer**	**heparin group**	**p17 amino acid**	**heparin group**	**p17 amino acid**
**IdoA 1**	**A**	2-O-SO_3^-<br>5-O_{hc}<br>6-COO^-	R_{22}<br>R_{22}<br>R_{22}	n.d.	n.d.
**Glc 2**		N-SO_3^-<br>6-O-SO_3^-	K_{27}<br>R_{22}	6-O-SO_3^-	R_{22}, K_{26}*
**IdoA 3**		2-O-SO_3^-<br>3-OH	K_{30}<br>K_{32}	2-O-SO_3^-<br>3-OH	K_{27}<br>K_{30}, K_{30}
**Glc 4**		5-O_{hc}<br>6-O-SO_3^-	K_{32}<br>K_{32}	6-O-SO_3^-	K_{27}, K_{30}, K_{32}, H_{33}
**IdoA 5**		2-O-SO_3^-	R_{39}	2-O-SO_3^-<br>3-OH	R_{39}<br>K_{32}
**Glc 6**		6-O-SO_3^-	R_{39}	N-SO_3^-<br>3-OH	R_{43}<br>R_{39}
**IdoA 7**		n.d.	n.d.	5-O_{hc}<br>6- COO^-	R_{43}<br>R_{39}
**Glc 8**	**B**	3-OH<br>N-SO_3^-	R_{43}<br>R_{43}	N-SO_3^-	R_{39}, R_{43}
**IdoA 9**		n.d.	n.d.	n.d.	n.d.
**Glc 10**		N-SO_3^-<br>6-O-SO_3^-	R_{39}<br>W_{36}	6-O-SO_3^-	K_{32}
**IdoA 11**		6- COO^-	K_{32}	6- COO^-	K_{32}
**Glc 12**		n.d.	n.d.	6-O-SO_3^-	K_{30}
**IdoA 13**		3-OH	K_{32}	2-O-SO_3^-	K_{27}, K_{30}, K_{32}
**Glc 14**		N-SO_3^-	K_{27}	N-SO_3^-	K_{27}
**IdoA 15**		2-O-SO_3^-	K_{30}	2-O-SO_3^-	K_{26}, K_{28}, K_{30}

Suppl. Tab. 2: H-bonds analysis of the 24-mer heparin/p17 dimer complex from docking or MDs. The negatively charged SO_3^- of the 24-mer heparin create a stable network of interactions with monomer A: [R_{22} (99%), K_{26} (98%), K_{27} (99%), K_{28} (80%) of the N-HBDs, R_{22} (99%), K_{27} (99%), Q_{65} (75%), Q_{69} (80%), R_{76} (75%) of the globular domain, Q_{65} (75%), K_{98} (89%), K_{112} (96%), K_{114} (80%) of the C-terminus] and with monomer B [R_{22} (>99%), K_{27} (>99%) , K_{28} (70%), K_{30} (95%), K_{32} (75%) of N-HBDs, Q_{69} (65%), R_{76} (85%), S_{77} (85%), N_{80} (99%) of the globular domain and K_{98} (96%), K_{112} (98%), K_{114} (90%) of the C-terminus]. In brackets are the average persistency of the different H-bonds. P17 amino acids in loop 1 are in red, those in the N- or C-HBD in blue, those in the α-helix 2, 3, 4 and 5 in green, violet, cyan and brown, respectively. IdoA: 2-O-sulfated L-iduronic acid. Glc: N, 6-O-disulfate D-glucosamine. Hc: heterocyclic. n.d.: not defined. H-bond with heparin is mediated by the N (*) or O (**) atom of the peptide bond. In all the other cases, N atoms of the side chain is instead involved.

		docking		MDs	
**heparin unit**	**p17 monomer**	**heparin group**	**p17 amino acid**	**heparin group**	**p17 amino acid**
**IdoA 1**	**A**	2-O-SO_3^-	K_{28}**, K_{30}	2-O-SO_3^-	K_{28}**
**Glc 2**		N-SO_3^-<br>6-O-SO_3^-	K_{26}<br>K_{27}	n.d.	n.d.
**IdoA 3**		2-O-SO_3^-	K_{26}	3-OH<br>6-COO^-	K_{26}<br>K_{27}
**Glc 4**		6-O-SO_3^-	R_{22}	6-O-SO_3^-	R_{22}
**IdoA 5**		n.d.	n.d.	2-O-SO_3^-	R_{22}
**Glc 6**		6-O-SO_3^-	K_{98}	N-SO_3^-	K_{98}
**IdoA 7**		2-O-SO_3^-	R_{76}<br>N_{80}	5-O_{hc}<br>6-COO^-	K_{98}<br>K_{98}
**Glc 8**		6-O-SO_3^-	Q_{69}	6-O-SO_3^-	N_{80}
**IdoA 9**		3-OH	Q_{65}	2-O-SO_3^-<br>5-O_{hc}<br>6-COO^-	R_{76}<br>K_{112}<br>K_{112}
**Glc 10**		N-SO_3^-	Q_{69}	1-OH<br>N-SO_3^-<br>6-O-SO_3^-	K_{112}<br>R_{76}<br>Q_{69}
**IdoA 11**	**A and B**	2-O-SO_3^-	Q_{65} (monomer B)	5-O_{hc}<br>6-COO^-	K_{114}*<br>K_{114} (monomers A and B)
**Glc 12**		N-SO_3^-	R_{58} (monomer B)	N-SO_3^-<br>6-O-SO_3^-	K_{114} (monomer B)<br>A_{115}*, Q_{116}* (monomer A)
**IdoA 13**	**B**	6-COO^-	Q_{108}	5-O_{hc}<br>6-COO^-	K_{112}<br>Q_{65}, Q_{69}, K_{112}
**Glc 14**		N-SO_3^-	K_{112}	1-OH<br>N-SO_3^-<br>4-OH	K_{112}<br>K_{112}<br>Q_{69}
**IdoA 15**		2-O-SO_3^-<br>3-OH	E_{105}*<br>Q_{65}	2-O-SO_3^-<br>3-OH	Q_{65}, Q_{69}<br>Q_{69}
**Glc 16**		N-SO_3^-<br>6-O-SO_3^-	N_{80}<br>Q_{69}, R_{76}	N-SO_3^-<br>3-OH	R_{76}, K_{98}<br>K_{98}
**IdoA 17**		n.d.	n.d.	2-O-SO_3^-<br>6-COO^-	R_{22}, N_{80}<br>K_{98}

**Glc 18**		N-SO_3^-<br>6-O-SO_3^-	R_{76}<br>R_{22}	1-OH	R_{22}
**IdoA 19**		2-O-SO_3^-<br>3-OH<br>5-O_{hc}	N_{80}<br>E_{73}**<br>R_{22}	2-O-SO_3^-	R_{22}, S_{77}**, N_{80}
**Glc 20**		N-SO_3^-<br>6-O-SO_3^-	K_{27}<br>H_{33}	N-SO_3^-	K_{27}
**IdoA 21**		2-O-SO_3^-<br>5-O_{hc}<br>6-O-SO_3^-	K_{30}<br>K_{27}<br>K_{27}	2-O-SO_3^-<br>5-O_{hc}<br>6-O-SO_3^-	K_{30}, K_{32}<br>K_{27}<br>K_{27}
**Glc 22**		1-OH<br>2-O-SO_3^-<br>6-O-SO_3^-	K_{30}<br>K_{32}<br>K_{27}	3-OH	K_{30}
**IdoA 23**		6-COO^-	K_{30}	2-O-SO_3^-<br>6-COO^-	K_{26}<br>K_{28}
**Glc 24**		N-SO_3^-<br>6-O-SO_3^-	K_{28}<br>K_{26}	6-O-SO_3^-	R_{22}, K_{28}
