# Supplementary material for: Heparin and heparan sulfate proteoglycans promote HIV-1 p17 matrix protein oligomerization: computational, biochemical and biological implications
Source: Sci Rep. 2019 Oct 31;9:15768. doi: 10.1038/s41598-019-52201-w (PMC6823450; doi:10.1038/s41598-019-52201-w)

Heparin and heparan sulfate proteoglycans promotes HIV-1 p17 matrix protein oligomerization: computational, biochemical and biological implications.

Antonella Bugatti, Giulia Paiardi, Chiara Urbinati, Paola Chiodelli, Alessandro Orro, Matteo Uggeri, Luciano Milanesi, Arnaldo Caruso, Francesca Caccuri, Pasqualina D'Ursi and Marco Rusnati

SUPPLEMENTARY FULL-LENGTH BLOTS

Fig. 1d, original blot: the part of the blot indicated by the box has been flipped and used for the Figure.

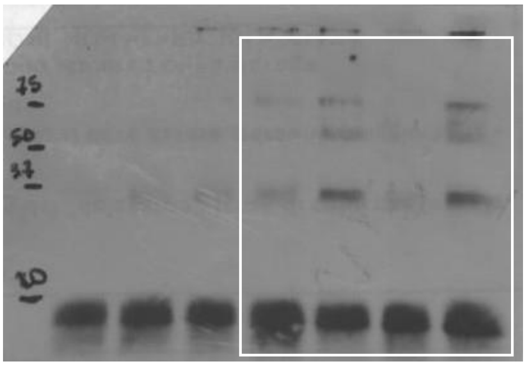

Fig. 2a, original blot: **t**he part of the blot indicated by the box has been used for the Figure.

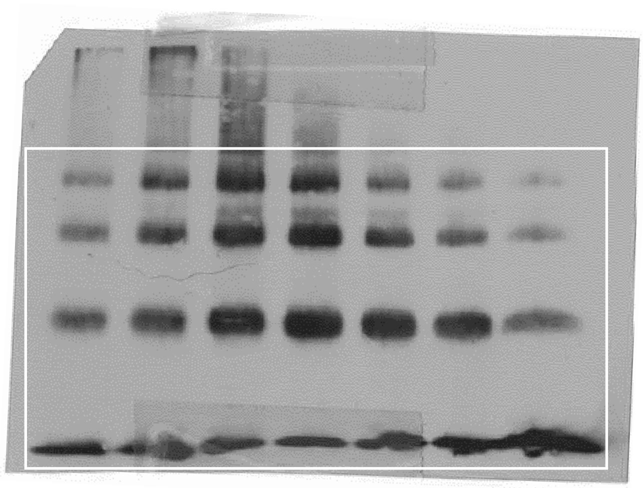

Fig. 3b, original blot: the parts of the blot indicated by the boxes have been cropped and used for the Figure.

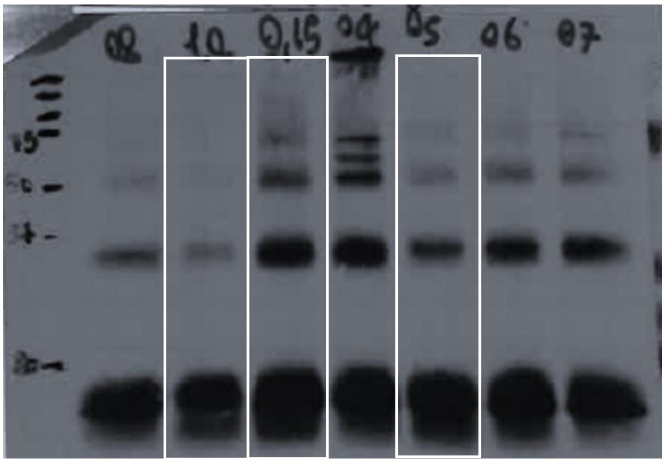

Inset of Fig. 4b, original blot: the part of the blot indicated by the box has been used for the inset.

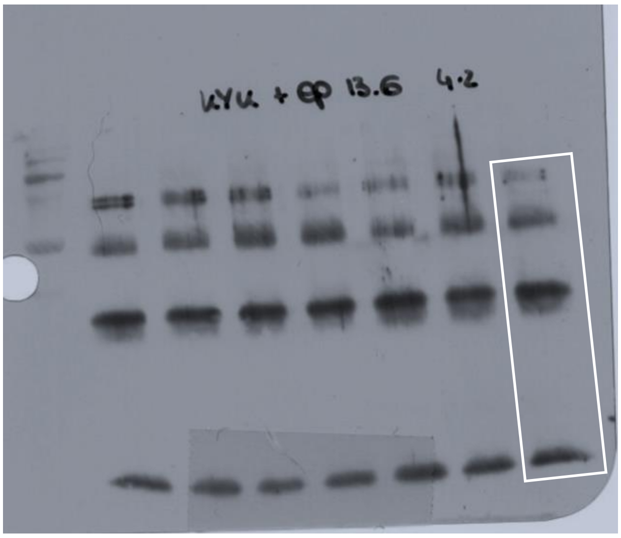

Fig. 7, original blot: the part of the blot indicated by the box has been used for the figure.

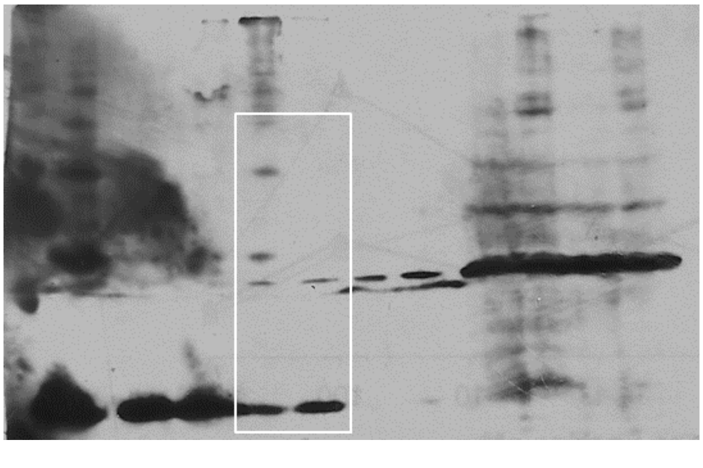

Fig. 8a, original blots: the analyses were performed on two separate gels for cells treated with chlorate (left) or left untreated (right). After protein transfer, the membranes were incubated with anti-$ERK_{1/2}$ and anti-FAK antibodies simultaneously. T→he parts of the blot containing the bands corresponding to the two antigens (indicated by the boxes) has been cropped and used for the figure.

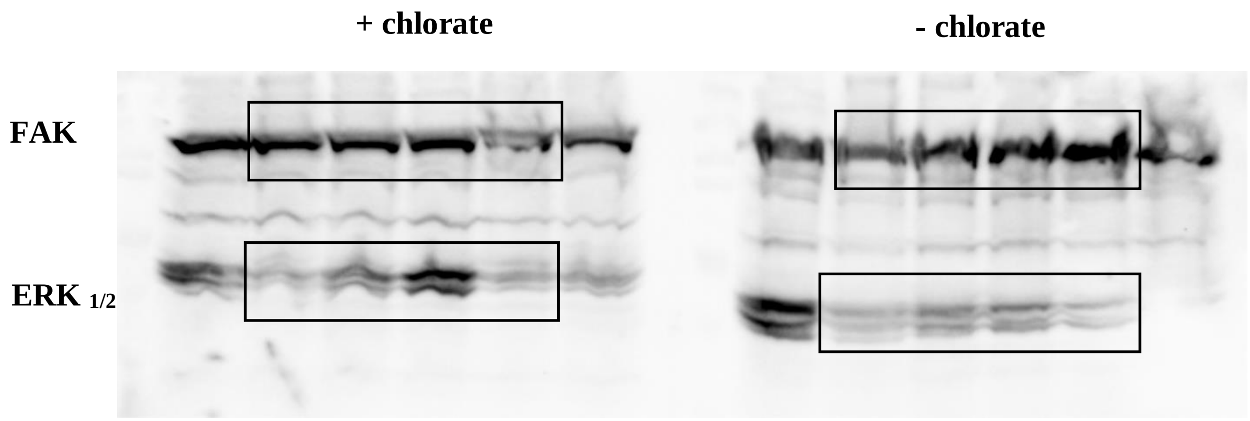

Fig. 8c, original blots: the analyses were performed on a single gel. After protein transfer, the membrane was cut to separate high molecular weight (containing FAK) and low molecular weight (containing $ERK_{1/2}$). Arrow point to the cut of the membrane. The two halves of the membrane were then incubated with anti-FAK and anti-ERK 1/2 antibodies, respectively. The parts of the blot containing the bands corresponding to the two antigens (indicated by the boxes) has been then cropped and used for the figure.

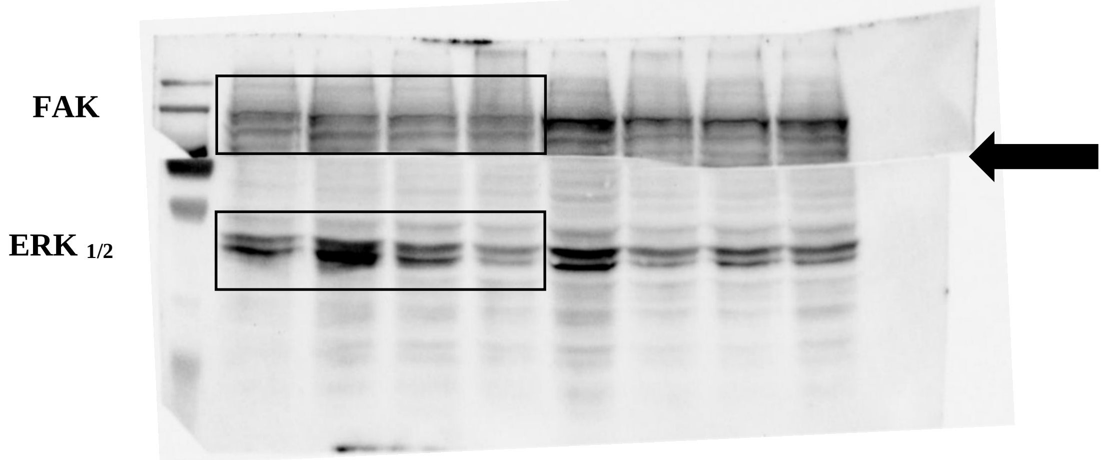

Supplement: Supplementary file 2 — original blots [file 41598_2019_52201_MOESM2_ESM.pdf]
